# Reversion from basal histone H4 hypoacetylation at the replication fork increases DNA damage in *FANCA* deficient cells

**DOI:** 10.1371/journal.pone.0298032

**Published:** 2024-05-31

**Authors:** Benilde García-de Teresa, Cecilia Ayala-Zambrano, Mirna González-Suárez, Bertha Molina, Leda Torres, Alfredo Rodríguez, Sara Frías

**Affiliations:** 1 Laboratorio de Citogenética, Instituto Nacional de Pediatría, Mexico City, Ciudad de México, Mexico; 2 Doctorado en Ciencias Biomédicas, Universidad Nacional Autónoma de México, Mexico City, Ciudad de México, Mexico; 3 Doctorado en Ciencias Biológicas, Universidad Nacional Autónoma de México, Mexico City, Ciudad de México, Mexico; 4 Laboratorio de Falla Medular y Carcinogénesis, Unidad de Genética de la Nutrición, Instituto Nacional de Pediatría, Mexico City, Ciudad de México, Mexico; 5 Departamento de Medicina Genómica y Toxicología Ambiental, Instituto de Investigaciones Biomédicas, Universidad Nacional Autónoma de México (UNAM), Mexico City, Ciudad de México, Mexico; Columbia University Irving Medical Center, UNITED STATES

## Abstract

The FA/BRCA pathway safeguards DNA replication by repairing interstrand crosslinks (ICL) and maintaining replication fork stability. Chromatin structure, which is in part regulated by histones posttranslational modifications (PTMs), has a role in maintaining genomic integrity through stabilization of the DNA replication fork and promotion of DNA repair. An appropriate balance of PTMs, especially acetylation of histones H4 in nascent chromatin, is required to preserve a stable DNA replication fork. To evaluate the acetylation status of histone H4 at the replication fork of *FANCA* deficient cells, we compared histone acetylation status at the DNA replication fork of isogenic *FANCA* deficient and *FANCA* proficient cell lines by using accelerated native immunoprecipitation of nascent DNA (aniPOND) and *in situ* protein interactions in the replication fork (SIRF) assays. We found basal hypoacetylation of multiple residues of histone H4 in FA replication forks, together with increased levels of Histone Deacetylase 1 (HDAC1). Interestingly, high-dose short-term treatment with mitomycin C (MMC) had no effect over H4 acetylation abundance at the replication fork. However, chemical inhibition of histone deacetylases (HDAC) with Suberoylanilide hydroxamic acid (SAHA) induced acetylation of the *FANCA* deficient DNA replication forks to levels comparable to their isogenic control counterparts. This forced permanence of acetylation impacted FA cells homeostasis by inducing DNA damage and promoting G2 cell cycle arrest. Altogether, this caused reduced RAD51 foci formation and increased markers of replication stress, including phospho-RPA-S33. Hypoacetylation of the *FANCA* deficient replication fork, is part of the cellular phenotype, the perturbation of this feature by agents that prevent deacetylation, such as SAHA, have a deleterious effect over the delicate equilibrium they have reached to perdure despite a defective FA/BRCA pathway.

## Introduction

DNA replication and repair occur in the context of chromatin. The basic unit of this dynamic nucleoprotein structure is the nucleosome, which is formed by 147 DNA base-pairs wrapped around a histone octamer composed by an H3-H4 tetramer and two H2A-H2B dimmers [1]. Post-translational modifications (PTMs) of histones participate in the fine-tuning modulation of chromatin structure to allow accurate cellular functions, by either modifying the union affinity of the histone octamer and the DNA molecule or by serving as docking stations for factors that regulate cellular processes [2].

Along with DNA, chromatin undergoes duplication, during the S phase of the cell cycle, a process that requires genome-wide disruption and restoration of chromatin structure: parental histones have to be evicted to allow the replication machinery to duplicate the DNA and once this has happened, a combination of newly synthesized histones and recycled parental histones are deposited onto the nascent DNA [3]. These newly synthesized histones have PTMs patterns that are highly conserved among eukaryotes and are rich in acetylation [4].

Chromatin duplication is a highly complex process that requires the coordinated participation of several proteins in the form of replisomes. This elaborated procedure can encounter obstacles like DNA damage, that generate replicative stress, for which cells have evolved mechanisms that process DNA damage in a replication-coupled manner in order to overcome these challenges and maintain genomic stability [5]. Appropriate replication requires a functional FA/BRCA pathway, a specialized repair system that acts during the S phase and consists of at least 22 gene products that coordinate to recognize and repair interstrand crosslinks (ICLs) and guard replication fork stability [6]. Constitutive loss of function of any of the FANC proteins results in a chromosome instability syndrome known as Fanconi anemia (FA), an inherited bone marrow failure and cancer predisposition syndrome that is characterized by hypersensitivity to agents that cause ICLs, like mitomycin C (MMC) or diepoxybutane (DEB) [6]. The latter is due to an inability to properly repair ICLs that results in DNA breakage and chromosomal rearrangements, a cellular phenotype that is sought in the chromosome fragility test, the gold standard for FA diagnosis [7]. FA derived cells are a natural occurring model of replication fork instability and replicative damage [8].

The state of the chromatin, regulated by PTMs, is relevant at the replication fork since it has a role in maintaining genomic integrity through stabilization of the replication fork and, when encountering replication blocking lesions, promoting DNA repair. Several particular PTMs have been shown to be important players contributing to genomic stability [9], but histone acetylation appears to be a particularly relevant one [10], especially since it is a druggable target that is promising for cancer treatment. It has been shown that histone acetylation at the replication fork must be tightly regulated, newly synthesized histones that will be deposited in the nascent DNA molecule have a highly conserved and characteristic di-acetylation pattern in lysine 5 and lysine 12 of histone H4, that is lost as the chromatin moves away from the fork [11].

Importantly, enzymatic machinery that modifies histone acetylation is known to travel with the replication fork and has a crucial role in its stability. The absence of acetyl eraser HDAC1/2 enzymes cause replication fork stalling [12], and deficiency of the acetyl writer Hat1 enzyme in mouse embryonic fibroblasts, results in growth defects, increased DNA damage and genome instability [13]. Moreover, acetylation of histones at the replication fork alleviates replication stress and facilitates DNA repair, directly linking histone acetylation at the fork with replication fork protection [14]. All this led us to ask if deviations in the balance of replication fork histone acetylation in FA cells contribute to genome instability.

Although it has been established that chromatin state is determinant for activation of the FA/BRCA pathway [15], there is scarce information regarding replication fork chromatin states of FA cells. In this study we explored the acetylation status of FA cells finding that FANCA deficient cells have hypoacetylated replication forks and that reverting this status by chemically compelling their acetylation causes DNA damage.

## Results

### Histone 4 is hypoacetylated in the replication fork of Fanconi anemia cells

Replication stress and activation of the DNA damage response have been observed after increased histone acetylation [16], and in the absence of histone acetyl transferase 1 (HAT1) or an opposing function enzyme, histone deacetylase 1 (HDAC1) [17, 18]. Since FA cells are characterized by deficient DNA repair and replication stress, we explored the acetylation status of FA replication forks and its connection to DNA damage.

**Fig 1:**
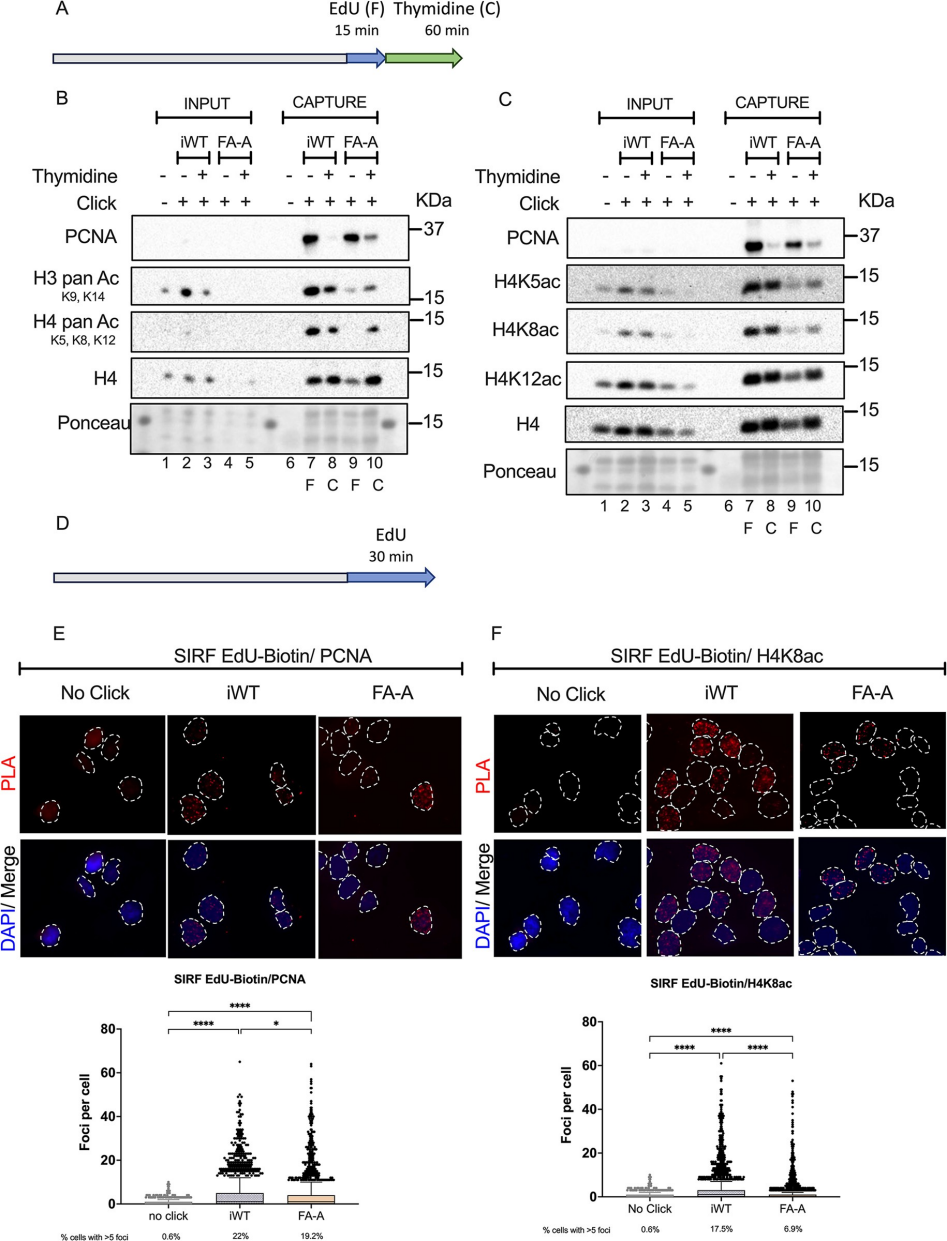
Histone 4 is hypoacetylated in the replication fork of FANCA deficient cells. **(A)** For aniPOND assays, cells were seeded and labeled the next day (grey bar) with an EdU [10 μM] pulse the last 15 min of culture (blue arrow), harvested immediately or chased in thymidine for 1 hour before harvest (green arrow). The cells were then clicked to biotin-azide before lysis and incubated overnight with streptavidin beads to precipitate EdU-associated proteins. **(B)** Representative immunoblots after capture of the replication fork using aniPOND in wild type and FA cells. Lanes 1 to 5 total protein of INPUT samples: lane 1 no click control, iWT cells in lanes 2 (pulse) and 3 (pulse and chase), FA-A cells in lanes 4 (pulse) and 5 (pulse and chase). Lanes 6 to 10 CAPTURE samples: lane 6 no click control, iWT cells in lanes 7 (pulse) and 8 (pulse and chase), FA-A cells in lanes 9 (pulse) and 10 (pulse and chase). All blots were performed in the same membrane for which a section of the Ponceau dye is shown. Blots show pan-acetylation of histone H3 and H4 in the iWT replication fork in comparison to FA-A cells. Cells were treated as in Fig 1A. F: capture of replication fork, C: capture of mature chromatin. **(C)** Representative immunoblots after capture of the replication fork using aniPOND in iWT and FA-A cells. Lanes 1 to 5 total protein of INPUT samples: lane 1 no click control, iWT cells in lanes 2 (pulse) and 3 (pulse and chase), FA-A cells in lanes 4 (pulse) and 5 (pulse and chase). Lanes 6 to 10 CAPTURE samples: lane 6 no click control, iWT cells in lanes 7 (pulse) and 8 (pulse and chase), FA-A cells in lanes 9 (pulse) and 10 (pulse and chase). All blots were performed in the same membrane for which a section of the Ponceau dye is shown. Blots show reduced acetylation of specific H4 residues: H4K5ac, H4K8ac and H4K12ac. Cells were treated as in Fig 1A. F: capture of replication fork, C: capture of mature chromatin. **(D)** For SIRF assays, cells were seeded in coverslips (grey bar) and labeled the next day with EdU [10 μM] for 30 min and fixed (blue arrow), EdU was then clicked to biotin azide before performing a PLA. **(E)** Representative images of a SIRF assay showing PLA foci of the interaction between EdU-Biotin and PCNA (*top*) dotted lines mark the nuclei. Quantification of the PLA interaction foci per cell from three independent replicates (*bottom*), a no click sample was included to ascertain PLA background signal. Cells were treated as in Fig 1D. Between 700 and 1000 cells per condition per experiment were analyzed. Differences were probed using the Kruskal Wallis test with Dunn’s post-test for multiple comparisons. **(F)** Representative images of a SIRF assay showing PLA foci of the interaction between EdU-biotin and H4K8ac (*top*), dotted lines mark the nuclei. Quantification of the PLA interaction foci per cell from three independent replicates (*bottom*), a no click sample was included to ascertain PLA background signal. Cells were treated as in Fig 1D. Between 700 and 1000 cells per condition per experiment were analyzed. Differences were probed using the Kruskal Wallis test with Dunn’s post-test for multiple comparisons. ****p<0.0001; *0.0332. Data are represented as mean ± SEM.

First, using aniPOND we analyzed the unperturbed replication fork of FANCA deficient GM6914+EV cells (FA-A), and its isogenic corrected counterpart GM6914+A (iWT). We compared the replication fork during a 15 min EdU pulse (F: fork) against the mature chromatin chased for 1 h in thymidine containing media after the EdU pulse (C: chromatin) (Fig 1A).

First, we confirmed that PCNA, a prototype protein of the replication fork, is unloaded from the chromatin once it moves away from the replication fork (Fig 1B; lane 8 and 10). Interestingly, in FA-A cells, PCNA can still be found in the one hour thymidine chase chromatin (Fig 1B; lane 10), reminding the pattern of incomplete PCNA unloading that has been observed in Hydroxyurea (HU) stalled replication forks from wild type cells [11] or indicating a basal slowed-down FA replication fork. Acetylation status analysis of the histones associated to the replication fork revealed pan-hypoacetylation of histones H3 and H4 (Fig 1B; lane 9) in a manner that is notable at the FA-A cells’ replication forks since histone acetylation levels are recovered once the chromatin matures (Fig 1B; lane 10). Examination of specific H4 lysine residues, the histone more intimately associated to DNA damage response [10], confirmed generalized H4 hypoacetylation and showed that H4K8 was the more affected residue (Fig 1C; lane 9).

The aniPOND assay is a global strategy that evaluates all the replication forks present in a cell culture at a given time [19]. Therefore, to have a better resolution of what was happening at individual forks in individual cells, we performed the SIRF assay, where the interaction of specific proteins with the newly synthesized DNA at the replication fork is assessed (Fig 1D). We probed PCNA interaction with newly synthesized DNA (EdU-Biotin label) and identified that FA-A cells had a smaller proportion of cells with PCNA interactions at the fork (Fig 1E). Moreover, the SIRF assay confirmed that FA-A cells had significantly lower interactions of acetylated histone H4K8 at the replication fork (Fig 1F). This was also evaluated in FANCG deficient cells, where we did not find this difference (S1 Fig). Together, the SIRF assays performed in FA-A cells, support what was previously seen by aniPOND.

### HDAC1 is enriched in the replication fork of FANCA deficient cells

**Fig 2:**
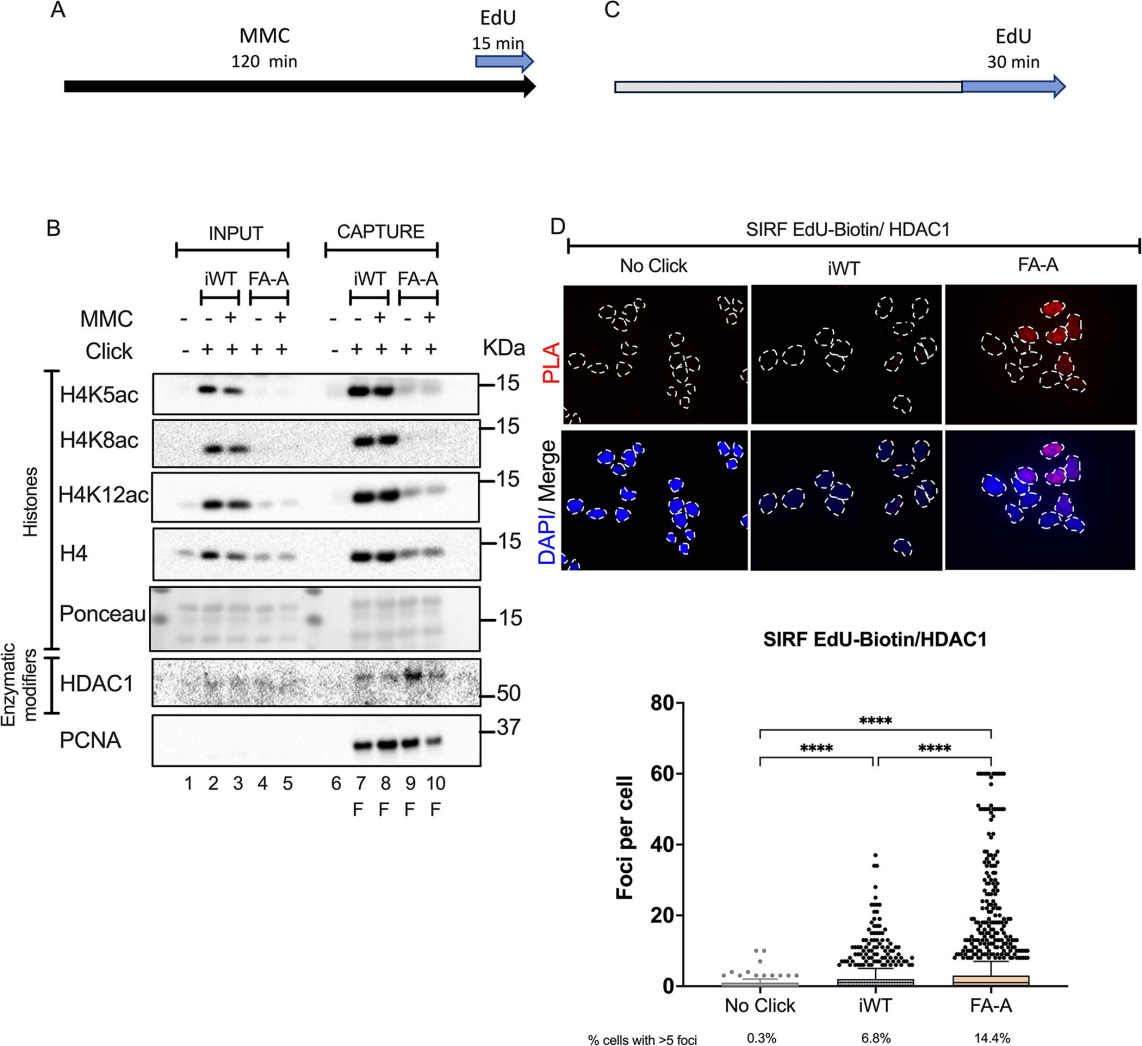
The histone deacetylase HDAC1 is enriched at the replication forks of FANCA deficient cells. **(A)** For aniPOND assays, cells were treated with MMC [1 μM] for 2 hours or left untreated (black arrow), labeled with an EdU pulse the last 15 min of culture (blue arrow), and harvested. **(B)** Representative immunoblots after capture of the replication fork of MMC-treated iWT and FA-A cells. Lanes 1 to 5 total protein of INPUT samples: lane 1 no click control, iWT cells in lanes 2 (pulse) and 3 (MMC and pulse), FA-A cells in lanes 4 (pulse) and 5 (MMC and pulse). Lanes 6 to 10 CAPTURE samples: lane 6 no click control, iWT cells in lanes 7 (pulse) and 8 (MMC and pulse), FA-A cells in lanes 9 (pulse) and 10 (MMC and pulse). All blots were performed in the same membrane for which a section of the Ponceau dye is shown. Blots show that MMC treatment does not change the reduced acetylation of histone H4 residues of FA cells (*top*). Blots show the presence of histone deacetylase 1 (HDAC1) (*bottom*). Cells were treated as in Fig 2A. F: capture of replication fork. **(C)** For SIRF assay, cells were seeded in coverslips and labeled the next day (grey bar) with EdU [10 μM] for 30 min and fixed (blue arrow), EdU was then clicked to biotin azide before performing a PLA. **(D)** Representative images of a SIRF assay showing PLA foci of the interaction between EdU-biotin and HDAC1 (*top*), dotted lines mark the nuclei. Quantification of PLA interaction foci per cell from three independent replicates (*bottom*), a no click sample was included to ascertain PLA background signal. Cells were treated as in Fig 2C. At least 500 cells per condition per experiment were analyzed. Differences were probed using the Kruskal Wallis test with Dunn’s post-test for multiple comparisons. ****p<0.0001. Data are represented as mean ± SEM.

Cells deficient in the FA/BRCA pathway are sensitive to ICL inducing agents. This pathway is activated upon recognition of the branched structure resulting from a stalled replication fork when it encounters an ICL [20]. To explore if H4 acetylation status was modified by a damage stimulus, we pulse-treated iWT and FA-A cells for 2 hours with a high dose of MMC [1 μM] (Fig 2A). Surprisingly, the acetylation status at the replication forks, as probed by aniPOND assay, was not modified by this treatment with MMC. The three probed H4 acetylated residues (K5, K8 and K12) showed the same pattern in the treated and untreated conditions (Fig 2B).

In light of the hypoacetylated H4 at the FANCA deficient replication fork, we probed the presence of acetylation machinery known to travel with the replication fork: eraser histone deacetylase HDAC1 [11, 16, 21] and writer HAT1 [18, 21]. Using the aniPOND cell population-based assay we found enrichment of HDAC1 at the FANCA deficient replication fork (Fig 2B and S2B Fig), a finding that was confirmed when looking at individual replication forks in the SIRF assay (Fig 2C). These results indicate a higher percentage of FA-A cells with HDAC1 at the replication fork when compared to iWT (Fig 2D). HAT1, a fork travelling writer responsible with acetylating all the probed residues, was found enriched at the FA-A replication fork when compared to iWT cells (S2D Fig).

### Induced acetylation increases DNA damage in FANCA deficient cells

**Fig 3:**
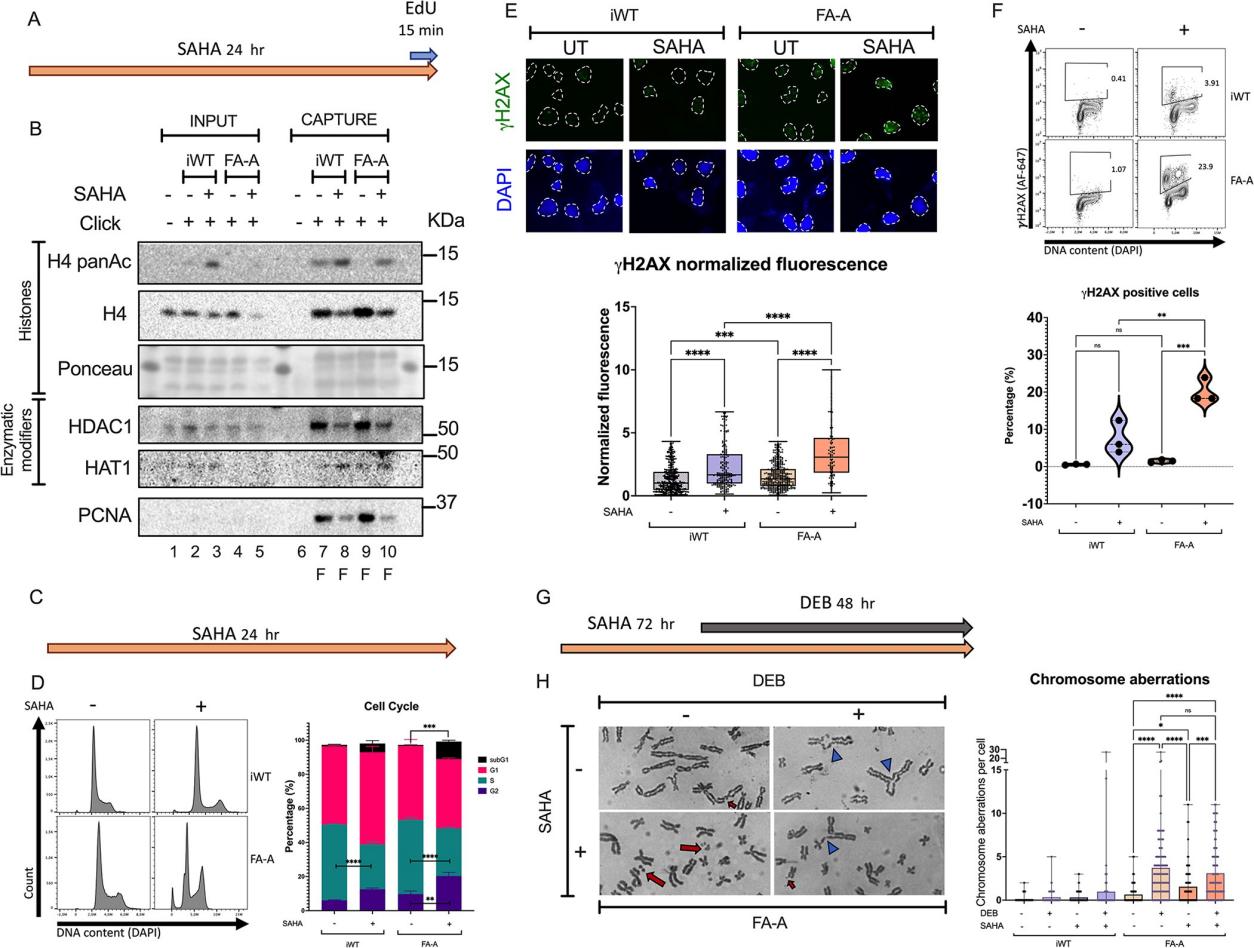
Induced acetylation of the replication fork increases DNA damage. **(A)** For aniPOND assays cells were treated with SAHA [1 μM] for 24 hours or left untreated (yellow arrow), labeled with an EdU [10 μM] pulse for 15 mins (blue arrow), and harvested. **(B)** Representative immunoblots after capture of the replication fork of SAHA treated wild type and FA cells. Lanes 1 to 5 total protein of INPUT samples: lane 1 no click control, iWT cells in lanes 2 (pulse) and 3 (SAHA and pulse), FA-A cells in lanes 4 (pulse) and 5 (SAHA and pulse). Lanes 6 to 10 CAPTURE samples: lane 6 no click control, iWT cells in lanes 7 (pulse) and 8 (SAHA and pulse), FA-A cells in lanes 9 (pulse) and 10 (SAHA and pulse). All blots were performed in the same membrane for which a section of the Ponceau dye is shown. Blots show that SAHA treatment increases acetylation of the replication fork of both cell lines and brings acetylation of the FA replication fork to unperturbed WT levels (*top*). Blots show that SAHA treatment decreases the presence of HDAC1 and PCNA, and increases the presence of HAT1 at the replication fork (*bottom*). Cells were treated as in Fig 3A. F: capture of replication fork. **(C)** For flow cytometry and immunofluorescence, cells were treated with SAHA [1 μM] for 24 hours or left untreated (yellow arrow), afterwards cells were harvested and stained accordingly. **(D)** Cells were stained with DAPI and DNA content was evaluated for cell cycle analysis. HDAC inhibition leads to an increase of the G2 phase and the sub-G1 population in both cell lines, although the FA pathway deficiency impacts to produce G2 arrest and impact cell viability. Cells were treated as in Fig 3C. Mean differences between each noted group were evaluated with the Student’s *t* test. **(E)** Representative images of γH2AX immunofluorescence (*top*) and quantification of one of five independent replicates showing an increase in γH2AX fluorescence upon HDAC inhibition in cells deficient for FANCA (*bottom*). Cells were treated as in Fig 3C, dotted lines mark the nuclei. Differences in the fluorescence of γH2AX among conditions were assessed using the Kruskall Wallis test with Dunn’s post-test for multiple comparisons. **(F)** Flow cytometry analysis of γH2AX+ cells (*top*). Cells were treated as in Fig 3C. Gating strategy showing that SAHA treatment leads to the induction of γH2AX. Proportion of γH2AX positive cells shows a significant increase from basal state in FA-A cells. Differences of three independent assays were probed using ANOVA and Tukey’s post-test for multiple comparisons. **(G)** For chromosome breakage analysis cells were pre-treated with SAHA [1 μM] for 24h (orange arrow) before inducing chromosomal aberrations with DEB [5 ng/mL] and the culture was continued for additional 48h (dark gray arrow). In cultures treated with SAHA the total time of exposure before harvest was 72 hours. **(H)** Metaphase spreads showing chromosome aberrations in response to SAHA and DEB in FANCA deficient cells: red arrows indicate breaks, and blue arrow tips show radial figures (*Left panel*). Quantification of chromosomal aberrations per cell shows a significant increase of chromosomal aberrations in FA cells when treated with DEB, SAHA or a combination of both agents (*Right panel*). Cells were treated as in Fig 3G. Three (iWT) or four (FA-A) independent experiments were performed. Differences between groups were evaluated using the Kruskal Wallis test with Dunn’s post-test for multiple comparisons. ****p<0.0001; ***p<0.0002; **0.0021; *0.0332; ns 0.1234.

HDAC1 chemical inhibition has been shown to increase histone acetylation at the replication fork [17], we therefore assessed the effect of inhibiting HDAC1 in FANCA deficient cells. We treated the cells with suberoylanilide hydroxamic acid (SAHA) and performed aniPOND (Fig 3A). This treatment resulted in H4 acetylation of the FA-A’s replication forks to levels comparable to iWT (Fig 3B). SAHA treatment also decreased the presence of HDAC1 at the replication fork as well as the abundance of PCNA in both cell lines, suggesting that HDAC inhibition reduces the number of cells in S phase (Fig 3B). For cell cycle analysis, cells were treated with SAHA for 24h (Fig 3C), the assay not only confirmed the reduction of S phase for both cell lines, but it also revealed a significant increase of FA cells in the sub-G1 and G2 cell cycle compartments (Fig 3D), mimicking the response of FA cells to ICL-inducing drugs [22].

SAHA has been shown to induce replicative DNA damage, likely by stalling the DNA replication forks [23]. When we evaluated γH2AX by immunofluorescence (Fig 3E) and by flow cytometry (Fig 3F), we evidenced a significant increase in DNA damage signaling in FA-A cells. In accordance with DNA damage induction, chromosome analysis of iWT and FA-A cells treated with SAHA (Fig 3G) showed an increase in the number of chromosome aberrations, though not as high as those in response to DEB induced ICLs. Notably, chromatid breaks, which are non-repaired DSB, were the most represented chromosomal aberration in response to SAHA (S3B and S3C Fig). Of note, a previous study looking at the effect of SAHA over the *in vitro* cellular FA phenotype [24] showed a reduction of chromosome radial figures after DEB treatment in patient-derived lymphocytes. In our system a median reduction of chromosomal aberrations of 15.87% (1.35%-52.56%) was observed, but this difference was not statistically significant (S3D-S3F Fig).

### Hyperacetylation induced by chemical HDAC inhibition stresses the replication forks of FANCA deficient cells

**Fig 4:**
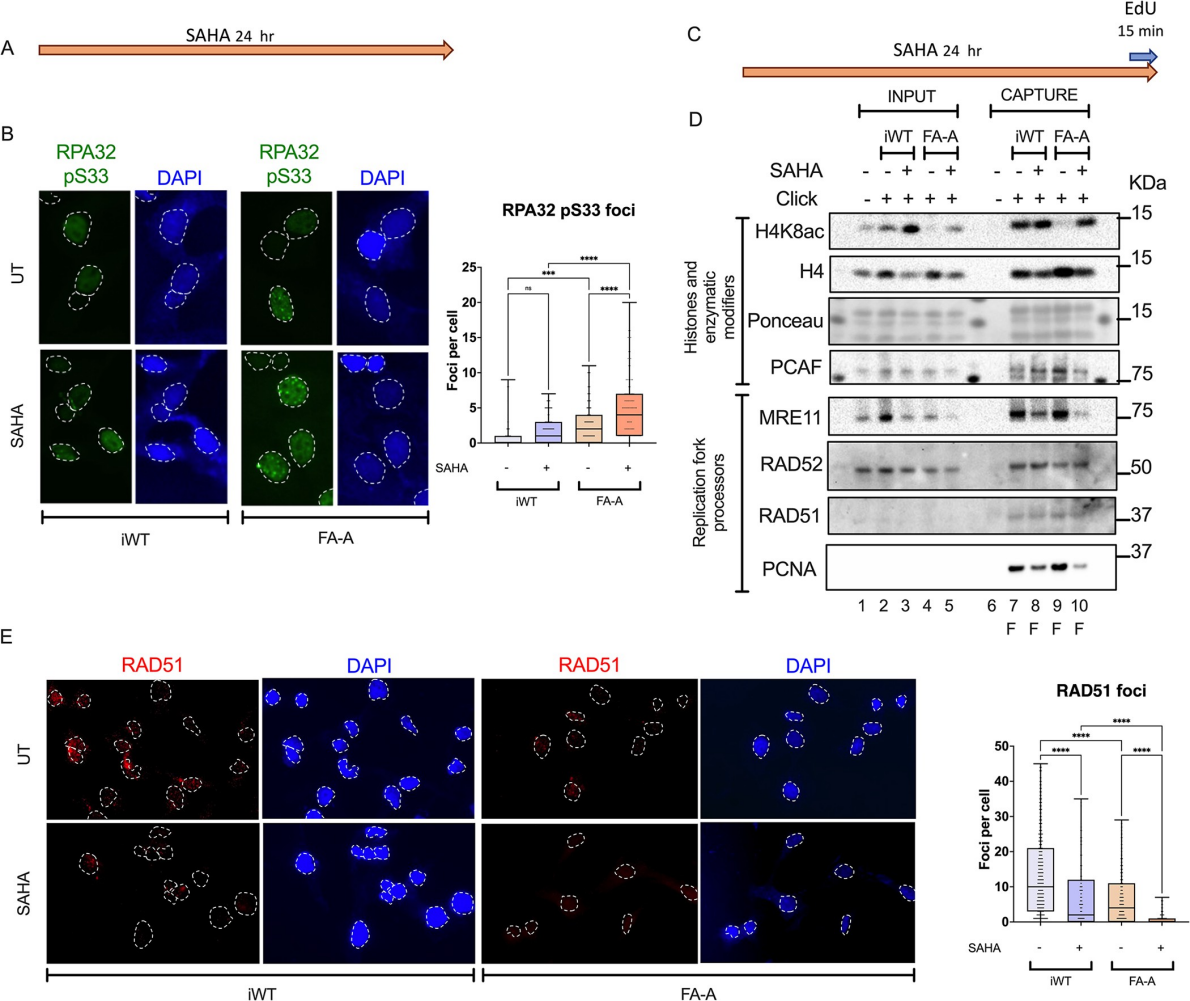
Hyperacetylation induced by chemical HDAC inhibition stresses the replication fork of FANCA deficient cells. **(A)** For immunofluorescence cells were treated with SAHA [1 μM] for 24 hours or left untreated (yellow arrow), cells were harvested and stained accordingly. **(B)** Illustrative images of RPA32-pS33 immunofluorescence (*left panel*) and quantification of one of three independent replicates showing an increase in RPA32-pS33 foci upon HDAC inhibition with SAHA in FA cells (*right panel*). Cells were treated as in Fig 4A, dotted lines mark the nuclei. Differences between conditions were assessed with the Kruskall-Wallis test with Dunn’s post-test for multiple comparisons. **(C)** For aniPOND and SIRF cells were treated with SAHA [1 μM] for 24 hours or left untreated (yellow arrow), labeled with an EdU [10uM] pulse for 15 mins for aniPOND or 30 min for SIRF (blue arrow) and harvested. **(D)** Representative immunoblots after capture of the replication fork of SAHA treated wild type and FA cells. Lanes 1 to 5 total protein of INPUT samples: lane 1 no click control, iWT cells in lanes 2 (pulse) and 3 (SAHA and pulse), FA-A cells in lanes 4 (pulse) and 5 (SAHA and pulse). Lanes 6 to 10 CAPTURE samples: lane 6 no click control, iWT cells in lanes 7 (pulse) and 8 (SAHA and pulse), FA-A cells in lanes 9 (pulse) and 10 (SAHA and pulse). All blots were performed in the same membrane for which a section of the Ponceau dye is shown. Blots show that SAHA treatment rescues H4K8 acetylation at the replication forks of both cell lines (*top*) and reduces the abundance of PCAF and MRE11 but does not affect the recruitment of RAD51 and RAD52 at the FA replication fork (*bottom*). Cells were treated as in Fig 4C. **(E)** Illustrative images of RAD51 immunofluorescence (left panel) and quantification of one of three independent replicates showing a decrease in RAD51 foci upon HDAC inhibition, an effect that is more dramatic in FA cells (*right panel*). Cells were treated as in Fig 4A. Differences between conditions were assessed using the Kruskall-Wallis test with Dunn’s post-test for multiple comparisons. ****p<0.0001; ***p<0.0002; **0.0021; *0.0332; ns 0.1234.

Given that SAHA produces replicative damage [23] and increases the amount of chromosome aberrations in FA-A cells, we looked for proxy signs of replication stress [25] after chemical inhibition of HDAC with SAHA (Fig 4A). The phosphorylation of serine 33 of RPA32 (RPA32-pS33) in FA-A cells was found to be increased in comparison to iWT (Fig 4B).

Using aniPOND we evaluated if SAHA treatment modified the recruitment of stress response and repair proteins to the replication fork (Fig 4C). From this assay we learned that MRE11, a nuclease responsible for fork degradation [26, 27] is present at both FA-A deficient and proficient replication forks. And that, SAHA treatment selectively decreased the abundance of MRE11 and PCAF, a histone acetyl transferase that mediates H4K8 acetylation to recruit MRE11 at stalled forks in BRCA deficient cells [28], without affecting other replication fork processors like RAD51 and RAD52 in FA-A cells (Fig 4D).

The way a cell responds to replicative stress is determined by the stimulus causing it as well as the available length of ssDNA to resolve the difficulty [29]. The effect of SAHA treatment does not appear to affect the ability to recruit RAD51 to the replication fork (Fig 4D), but the formation of RAD51 foci is compromised upon SAHA inhibition of HDAC since we observed a sharp decrease in RAD51 foci formation, more strikingly in FA-A cells (Fig 4E). This effect is reminiscent of the loss of FANCD2 and FANCI foci formation in response to SAHA in FA cells described by others [15].

## Discussion

In this study, we analyzed the acetylation status of the replication fork-associated histones of *FANCA* deficient cells and uncovered an unexpected feature: a reduction in the acetylation of histones associated to newly synthesized DNA in contrast to the high acetylation of wild type cells. We also found that inducing acetylation of the *FANCA* deficient fork, by inhibiting HDAC, recovered the acetylation pattern at the expense of producing replication stress and DNA damage.

DNA replication occurs in the context of chromatin, therefore chromatin structure and how it responds to stimuli occurring in the course of DNA synthesis is decisive for accurate replication [30]. The acetylation dynamics of newly deposited histones is a tightly regulated process: histones associated to newly synthesized DNA are highly acetylated and this mark is gradually lost when the maturing chromatin moves away from the replication fork [11]. This delicate balance is achieved by fine tuning the opposing functions of chromatin writer and eraser proteins that travel with the replication fork [11, 18].

Given that acetylation balance at the replication fork is crucial to maintain stability of the DNA molecules [16], it is of no surprise that altering its balance through chemical, epigenetic or genetic means has measurable effects. In this work, as in work by others [23], it has been shown that chemically inhibiting HDAC with SAHA affects chromatin structure, replication initiation and fork progression, leading to increased DNA damage as signaled by yH2AX (Fig 3E and 3F). In response to SAHA, the DNA damage response in *FANCA* deficient cells is altered and it translates into unrepaired DNA damage, as shown here in the form of chromosome aberrations (Fig 3G) that particularly consist of breaks (S3 Fig). Previous studies have shown that SAHA also induces DNA damage in normal cells, but these are able to repair the DNA damage [31].

In the cellular model that we used, hypoacetylation, notably affecting the replication fork-associated histones is part of the phenotype of FA-A cells (Fig 1). A similar observation has previously been presented in cells with siRNA-mediated FA pathway deficiency, showing a basal reduction of H4K16 acetylation [32]. The data presented here reveal that impaired acetylation in FA-A deficient cells have a wider reach and affect several residues. This suggests that hypoacetylation of the replication fork-associated histones is part of a rewiring mechanism of the FA replication fork with a certain composition that allows DNA replication despite basal increased replication stress levels. Or potentially a coping mechanism that allows FA-A cells to limit the formation of R-loops by down regulating transcription through hypoacetylation. In this way genome stability may be maintained by avoiding the formation of breaks due to the inability of FA deficient cells to process transcription-replication conflicts [33]. The fact that compelled histone acetylation induces breaks (S3 Fig) could be the result of eliminating the defense to R loop formation provided by histone hypoacetylation, this hypothesis may be worth exploring in the future.

Interestingly, modifying this feature, by inhibiting deacetylation to permit acetylation levels similar to the ones observed in the wild type cells results in DNA damage (Fig 3). These findings are in accordance to previous studies that have demonstrated that inhibiting HDAC with SAHA in cancer cell lines causes replication fork instability, and consequently, replicative stress and S phase reduction [23, 31, 34]. It is particularly interesting that our observation that MRE11 decreases at the replication forks of FANCA deficient cells in response to replication stress is a phenomenon previously described in FANCB [35] and BRCA2/FANCD1 [36] deficient cells, when exposed to mild replication stress. This observation coincides with an increase in RPA levels, as also shown here in FANCA cells (Fig 4). Myler has proposed a model in which resection is regulated by cyclic competition for the single stranded DNA by RPA and the exonucleases, allowing for limited resection on multiple cyclic rounds [37]. The decrease of MRE11 at the FANCA deficient replication forks in response to SAHA suggests that the replication stress, elicited by HDAC inhibition, is of a different kind than the one produced by high doses of HU where MRE11 accumulates at the replication fork [11]. This suggests that FANCA deficient cells may overcome this kind of stress by employing a different nuclease, echoing what others have suggested: different nucleases may act in a lesion-specific or context-specific manner [38]. It does not escape our attention that in our system, the localization of MRE11 to the replication fork is not dependent on the presence of H4K8ac (Fig 4D) as it has recently been shown [28]. These discordant results prompt the hypothesis that alternative signals can drive MRE11 to the replication fork, to bring the appropriate nuclease in a given context.

Replication fork instability is a cellular phenotype of FA/BRCA pathway deficient cells mainly studied in FA subgroups with known replication fork-associated functions (*FANCD1/BRCA1*, *FANCF/RAD51*, *FANCS/BRCA2*, *FANCU/XRCC2*, *FANCO/RAD51C* and *FANCN/PALB2*) that are classical HR factors which act in the downstream part of the FA/BRCA pathway [39]. Nevertheless, there are few studies that explore this in upstream FA/BRCA subgroups, like Schlacher’s key study, where she showed that *FANCA* deficient cells have increased DNA resection when exposed to HU [27].

Replication stress response at the replication fork is a very active area of research. The interplay between proteins with replication fork protection tasks and the nucleases that make them unstable, has proven to be an intricate network that is still being refined. Which nucleases are responsible for resection in different stress contexts and how this is regulated is an area that merits further investigation.

Our findings are important because HDAC inhibitors are currently being evaluated as part of treatment regimens for acute myeloblastic leukemia [40] and advanced stage head and neck squamous cell carcinoma [41]. Both of these conditions are frequent oncologic complications for patients with FA, for which treatment is really difficult [42, 43]. Transfer of therapeutic options to a FA context must contemplate cellular responses inherent to the FA/BRCA pathway defect. As new drug options are considered for cancer treatment, it is of utmost importance to keep in mind that in patients with FA, the characteristics that account for specific toxicity of HDAC inhibitors in cancer cells are present in all of the patient’s cells, rendering them more susceptible to toxicity.

### Limitations of the study

This study is limited by the fact that experiments were mainly conducted in a single pair of isogenic cell lines. There may be differences in the replication fork status of FA cells depending on the FA/BRCA pathway gene that is affected, as revealed by the analysis of FANCG cells (S1 Fig). Also, since we followed a hypothesis driven strategy, we only analyzed a subset of proteins present at the replication fork, and given that the cell has redundant systems that participate in protein acetylation, we may have missed other proteins that contribute to the hypoacetylation phenotype of these cells. Moreover, to evaluate the effect of induced replication fork acetylation, we used SAHA, a non-selective inhibitor of HDAC, although we achieved the desired histone acetylation at the replication fork to which we ascribe the damaging effects of inhibiting HDAC, we cannot rule out that there may also be a contribution of modifying acetylation status of other targets.

## Materials and methods

### Cell culture

Patient derived GM6914+A and GM6914+EV fibroblast cell lines, a generous gift from Dr. Alan D’Andrea (Dana Farber Cancer Institute, Boston MA, USA), were cultured in Dulbecco’s modified Eagle’s medium (Gibco^TM^ Thermo Fisher Scientific Cat#) with 10% fetal bovine serum (Gibco^TM^ Thermo Fisher Scientific Cat# 16000069), 200mM L-glutamine (Sigma-Aldrich Cat# G6392) and 1x Penicillin-Streptomycin solution (Gibco^TM^ Thermo Fisher Scientific Cat# 15140130).

### Resources

Key resources are itemized in Tables 1–5.

**Table 1:** Experimental model: Cell lines.

CELL LINE	SOURCE	IDENTIFIER
Human: GM6914+A	Dr. Alan D’Andrea’s Lab, DFCI	Näf et al. 1998 [44]
Human: GM6914+EV	Dr. Alan D’Andrea’s Lab, DFCI	Näf et al. 1998 [44]

**Table 2:** Antibodies.

ANTIBODY	SOURCE	IDENTIFIER
Anti FANCA Antibody (D1227)	Cell signaling	Cat# 14657S RRID:AB_2798558
Anti-PCNA Antibody (Rb)	Genetex	Cat# GTX100539 RRID:AB_1241163
Anti-PCNA Antibody (Ms)	Novus Biologics	Cat# NB500-106 RRID:AB_2252058
Anti-Acetyl H3K9/K14 Polyclonal Antibody	Epigenetek	Cat# A4021
Anti-H4ac pan acetyl (K5, K8, K12) Antibody	Invitrogen^TM^ Thermo Fisher Scientific	Cat# PA05-40083
Anti H4	Abcam	Cat# ab13843
Anti-H4K5ac Antibody	Epigenetek	Cat# 804415
Anti-H4K8ac Antibody	Genetex	Cat# GTX60906
Anti-H4K12ac Antibody	Epigenetek	Cat# 60392
Anti-Biotin Antibody	Sigma	Cat# SAB4200680
Anti-HDAC1 Antibody	Abcam	Cat# ab7028 RRID:AB_305705
Anti-KAT1/HAT1 Antibody	Abcam	Cat# ab194296 RRID:AB_2801641
Anti-γH2AX (pS139) Antibody	Abcam	Cat# ab81299 RRID:AB_1640564
Alexa Fluor™ 647 Mouse Anti-γH2AX (pS139) Antibody	BD	Cat# 51–9007683
Anti-RPA32 p33 Antibody	Abcam	Cat# ab211877 RRID:AB_2818947
Anti-KATB2/PCAF Antibody (G14G9)	Cell Signaling	Cat# 3378S RRID:AB_2128409
Anti-MRE11 Antibody	Cell Signaling	Cat# 4895 RRID:AB_2145100
Anti-RAD51 Antibody	Genetex	Cat# GTX70230 RRID:AB_372856
Anti-RAD52 Antibody	Santa Cruz	Cat# sc-365341 RRID:AB_10851346
Goat anti-Rabbit IgG (H+L) Cross-Adsorbed Secondary Antibody, Alexa Fluor^TM^488	Invitrogen^TM^ Thermo Fisher Scientific	Cat# A-11008 RRID:AB_143165
Goat anti-Mouse IgG (H+L) Cross-Adsorbed Secondary Antibody, Alexa Fluor^TM^555	Invitrogen^TM^ Thermo Fisher Scientific	Cat# A-21422 RRID:AB_2535844
Anti-rabbit IgG, HRP-linked Antibody	Cell Signaling	Cat# 7074 RRID:AB_2099233
Anti-mouse IgG, HRP-linked Antibody	Cell Signaling	Cat# 7076 RRID:AB_330924

**Table 3:** Chemicals.

CHEMICALS	SOURCE	IDENTIFIER
Suberoylanilide hydroxamic acid (SAHA)	Sigma-Aldrich	Cat# SML0061
Mitomycin C (MMC)	Sigma-Aldrich	Cat# 10107409001
1,3-Butadiene diepoxide (DEB)	Sigma-Aldrich	Cat# 202533
5-ethynyl—2’-deoxyuridine (EdU)	Invitrogen^TM^ Thermo Fisher Scientific	Cat# A10044
Thymidine	Sigma-Aldrich	Cat# T1895
Azide-PEG3-biotin conjugate	Sigma-Aldrich	Cat# 762024

**Table 4:** Critical commercial assays/reagents.

REAGENT	SOURCE	IDENTIFIER
Duolink® In Situ PLA® Probe Anti-Rabbit MINUS	Sigma-Aldrich	Cat# DUO92005
Duolink® In Situ PLA® Probe Anti-Mouse PLUS	Sigma-Aldrich	Cat# DUO92001
Duolink® In Situ Detection Reagents Red	Sigma-Aldrich	Cat# DUO92008
ECL^TM^ system Prime Western Blotting Detection Reagents	Amersham^TM^ GE	Cat# RPN2232
Cytofix/Cytoperm^TM^ Fixation/Permeabilization Solution	BD Biosciences	Cat# 51-2090KE
Streptavidin Magnetic Pearls Pierce^TM^	Thermo Fisher Scientific	Cat# 88817
DAPI	BD	Cat# 51–9007681
VECTASHIELD® Mounting Medium with DAPI	Vector Laboratories	Cat# H-1200
IGEPAL® CA-630	Sigma-Aldrich	Cat# I8896
10% Normal Goat Serum	Life technologies	Cat# 50062Z
Restore PLUS Western Blot Stripping Buffer	Thermo Fisher Scientific	Cat# 46430

**Table 5:** Software and algorithms.

SOFTWARE	SOURCE	IDENTIFIER
FIJI	Open source	RRID:SCR_002285 http://fiji.sc Schindelin et al. 2012 [45]
Prism (v10)	GraphPad	RRID:SCR_002798 https://www.graphpad.com
Zeiss Zen Lite	Zeiss group	RRID:SCR_023747 https://www.zeiss.com/microscopy/en/products/software/zeiss-zen-lite.html
ImageLab	BioRad	RRID:SCR_014210 http://www.bio-rad.com/en-us/sku/1709690-image-lab-software
FlowJo	BD Biosciences	RRID:SCR_008520 https://www.flowjo.com/solutions/flowjo

### aniPOND: Accelerated native immunoprecipitation of nascent DNA

The Modified aniPOND protocol by Weist [46] was used to identify proteins at replication forks. In brief, exponentially growing GM6914 cells were either not treated, treated with Mitomycin C (MMC) [1μM] or pretreated for 24h with SAHA [1mM] and pulsed the last 15 min with EdU [10μM]. Afterwards, nuclear extraction was performed with NEB buffer (HEPES [20mM], MgCl [3mM], sucrose [300 mM], NaCl [50 mM], and 0.5% IGEPAL CA630), after a PBS wash, nuclei were subjected to the click reaction (biotin azide [25μM], (+) sodium L ascorbate [10 mM], CuSO_4_ [2mM]) for 60min. Nuclei were washed once in PBS then rotated twice for 30 min in B1 buffer (NaCl [25 mM], EDTA [2 mM], Tris-HCl [50 mM] pH 8.0, 0.5% IGEPAL CA630 plus protease inhibitors). Afterwards, chromatin bound material was solubilized using a microtip sonicator by a total of 12 rounds of 10s on- 10s off with approximately 10W output. Clarified solubilized chromatin was immunoprecipitated overnight with streptavidin coated magnetic beads (Pierce). A total input aliquot (2% of total lysate) was taken from the lysate before overnight streptavidin capture. After 18-20h of capture, extensive washes (6–7) with B1 buffer were performed. Both input and capture samples were eluted with 2x Laemmli sample buffer with 5% β-mercaptoethanol by heating the samples to 95°C for 15 min. Samples were resolved in polyacrylamide gels and transferred onto nitrocellulose membranes. Membranes were blocked in 5% milk in TBS, and probed for protein detection with specific primary antibodies, secondary antibodies were used at a 1:10,000 dilution. Detection was done through chemiluminescence using the ECL system. Sequential queries were made in the same membrane by stripping it with Restore stripping buffer for 10 min followed by 2x TBS wash.

### Immunofluorescence

Cells were sequentially fixed with formaldehyde 2% for 10 min at RT then with methanol 100% for 20 min at -20°C, washed twice with PBS then permeabilized with triton 0.5% for 5 min, washed again twice with PBS before blocking with G serum at 37°C for 1 h. Primary antibodies were incubated overnight at 4°C. The following day, cells were washed three times with PBS, and incubated with fluorophore coupled secondary antibody at 37°C for 1 h. Finally, they were washed three times in PBS before mounting with Vectashield® with DAPI. Images were acquired with the 40x lens of a Zeiss Axio Imager Z1 using the Zen lite controller. Images were analyzed using ImageJ software for fluorescence intensity in each detected nucleus. At least 200 nuclei per condition per repetition were analyzed. The raw density measurements were normalized to an arbitrary value of 10 for the highest fluorescence intensity value.

### SIRF proximity ligation assay for protein interactions at the replication fork

Replicating cells were pulsed with EdU [10μM] 30 min to mark replication forks. Cells were washed twice with PBS, then fixed sequentially with formaldehyde 2% for 10 min at RT then methanol 100% for 20 min at 4°C, washed twice with PBS then permeabilized with triton 0.5% for 5 min, washed again twice with PBS before being subjected to the click reaction (biotin azide [25μM], (+) sodium L ascorbate [10 mM], CuSO_4_[2mM]) for 30 min in movement. Three washes with PBS were followed by a 1h block at 37°C using blocking solution (Thermo Fisher), then primary antibodies were incubated overnight at 4°C. The next day, *in situ* proximity ligation assay was performed using the Duolink Detection Kit and protocol (Sigma Aldrich Duolink). In brief, secondary antibodies bound to PLA probes were incubated for 1 hour at 37°C, then the ligation reaction was performed for 30 min at 37°C. Finally, an amplification reaction using a fluorescent probe was allowed to take place for 140 min at 37°C. Images were acquired with the 40x lens of a Zeiss Axio Imager Z1 using the Zen lite controller. Images were analyzed using ImageJ software, with foci count according to the protocol published by Lazarchuk [47], a threshold of 5 foci was determined as a positive biotin-protein interaction.

### Chromosomal aberrations

Cells were seeded either with SAHA [1 μM] or vehicle (ethanol), 24 hours after seeding half the cultures were treated with DEB 5 ng/mL for 48 hours. Two hours before completion of DEB treatment, colchicine was added to the cultures for 2 h. Cells were collected and fixed using Carnoy solution following standard protocol [7]. Suspension cells were spread on slides and stained with Giemsa. Twenty-five metaphases per culture were analyzed for chromosome aberrations, gaps were excluded.

### Flow cytometry

Cells were seeded either with SAHA [1 μM] or vehicle (ethanol), 24 hours after seeding, cells were harvested, fixed and permeabilized using the cytofix/cytoperm^TM^ fixation/permeabilization solution from BD according to manufacturer’s indications. Cells were then stained with a Mouse Anti- γH2AX Alexa Fluor™ 647 coupled antibody and DAPI. Samples were resolved in a Cytoflex instrument and analyzed with Flowjo software.

### Statistical analysis

All statistical analyses were performed using Prism software. Data distribution was first evaluated. The means of data with normal distributions were compared using One way ANOVA with Tukey’s post-test for multiple comparisons. When only two groups were compared, we used a student’s t test. And, for data without normal distributions, we used the Kruskal Wallis non parametric test with Dunn’s post-test for multiple comparisons. All numerical data are available in S1 File.

## Supporting information

S1 Fig **(A)** FANCG proficient PD326+G (iWT) and FANCG deficient PD326+EV (FA-G) cells were seeded in coverslips and labeled the next day (grey bar) with EdU [10 μM] for 30 min and fixed (blue arrow), EdU was then clicked to biotin azide before performing a PLA. **(B)** Representative images of a SIRF assay showing PLA foci of the interaction between EdU-Biotin and H4K8ac, dotted lines mark the nuclei. **(C)** Quantification of the PLA interaction foci per cell from one independent replicate, a no click sample was included to ascertain PLA background signal. Cells were treated as in S1A Fig. At least 200 cells per condition were analyzed. Differences were probed using the Kruskal Wallis test with Dunn’s post-test for multiple comparisons. ****p<0.0001; ns 0.1234. Data are represented as mean ± SEM.
(TIF)

S2 Fig **(A)** For aniPOND assays, cells were seeded and labeled the next day (grey bar) with an EdU [10 μM] pulse the last 15 min of culture (blue arrow), harvested immediately or chased in thymidine for 1 hour before harvest (green arrow). The cells were then clicked to biotin-azide before lysis and incubated overnight with streptavidin beads to precipitate EdU-associated proteins. **(B)** Representative immunoblots after capture of the replication fork in iWT and FA-A cells. Lanes 1 to 5 total protein of INPUT samples: lane 1 no click control, iWT cells in lanes 2 (pulse) and 3 (pulse and chase), FA-A cells in lanes 4 (pulse) and 5 (pulse and chase). Lanes 6 to 10 CAPTURE samples: lane 6 no click control, iWT cells in lanes 7 (pulse) and 8 (pulse and chase), FA-A cells in lanes 9 (pulse) and 10 (pulse and chase). All blots were performed in the same membrane for which a section of the Ponceau dye is shown. Blots show the presence of histone deacetylase 1 (HDAC1) and histone acetyl transferase 1 (HAT1). Cells were treated as in S2A Fig. F: capture of replication fork, C: capture of mature chromatin. **(C)** For SIRF assay, cells were seeded in coverslips and labeled the next day (grey bar) with EdU [10 μM] for 30 min and fixed (blue arrow), EdU was then clicked to biotin azide before performing a PLA. **(D)** Representative images of a SIRF assay showing PLA foci of the interaction between EdU-biotin and HAT1 (*top*), dotted lines mark the nuclei. Quantification of PLA interaction foci per cell from three independent replicates (*bottom*), a no click sample was included to ascertain PLA background signal. Cells were treated as in S2C Fig. At least 500 cells per condition per experiment were analyzed. Differences were probed using the Kruskal Wallis test with Dunn’s post-test for multiple comparisons. ****p<0.0001. Data are represented as mean ± SEM.
(TIF)

S3 Fig **(A)** For chromosome breakage analysis cells were pre-treated with SAHA [1 μM] for 24h (orange arrow) before inducing chromosomal aberrations with DEB [5 ng/mL] and the culture was continued for additional 48h (dark gray arrow). In cultures treated with SAHA the total time of exposure before harvest was 72 hours. **(B)** Metaphase spread with representative breaks, DNA damage that affects the continuity of the metaphasic chromosome affecting either single or both chromatids (*Left panel*). Quantification of chromosomal breaks per cell shows a significant increase of breaks in FA-A cells when treated with DEB, SAHA or a combination of both agents (*Right panel*). Cells were treated as in S3A Fig. Three (iWT) or four (FA-A) independent experiments were performed. Mean and range are depicted. Differences between groups were probed using the Kruskal Wallis test. ****p<0.0001; **0.0021; *0.0332; ns 0.1234. **(C)** Proportion of chromosomal breaks from total chromosomal aberrations. Mean and range of three (iWT) or four (FA-A) independent experiments are graphed. SAHA mainly induces breaks in FA-A cells. **(D)** Metaphase spread with representative joined aberrations, resulting from breaks that attach to an incorrect partner, particularly radial figures, the classic aberration in FA/BRCA deficient cells (*Left panel*). Quantification of joined chromosomal aberrations per cell shows an increase in joined aberrations upon exposure to DEB in FA cells (*Right panel)*. Cells were treated as in S3A Fig. Three (iWT) or four (FA-A) independent experiments were performed. Mean and range are depicted. Differences between groups were probed using the Kruskal Wallis test. ****p<0.0001; ***p<0.0002; ns 0.1234. **(E)** Proportion of joined chromosomal aberrations from total chromosomal aberrations. Mean and range of three (iWT) or four (FA-A) independent experiments are graphed. SAHA pre-treatment appears to reduce the proportion of joined aberrations induced by DEB. **(F)** Table showing median and range of chromosomal aberrations, as well as proportion of aberrations that belong to the break or joined type, according to experimental condition.
(TIF)

S1 File Numerical data. (XLSX)
