# Correction: Reversion from basal histone H4 hypoacetylation at the replication fork increases DNA damage in *FANCA* deficient cells

**DOI:** 10.1371/journal.pone.0315966

**Published:** 2024-12-12

**Authors:** Benilde García-de-Teresa, Cecilia Ayala-Zambrano, Mirna González-Suárez, Bertha Molina, Leda Torres, Alfredo Rodríguez, Sara Frías

The first author’s name is spelled incorrectly. The correct name is: Benilde García-de-Teresa. The correct citation is: García-de-Teresa B, Ayala-Zambrano C, González-Suárez M, Molina B, Torres L, Rodríguez A, et al. (2024) Reversion from basal histone H4 hypoacetylation at the replication fork increases DNA damage in *FANCA* deficient cells. PLOS ONE 19(5): e0298032. https://doi.org/10.1371/journal.pone.0298032.
