# Supplementary figures and images for: Reversion from basal histone H4 hypoacetylation at the replication fork increases DNA damage in FANCA deficient cells
Source: PLoS One. 2024 May 31;19(5):e0298032. doi: 10.1371/journal.pone.0298032 (PMC11142588; doi:10.1371/journal.pone.0298032)

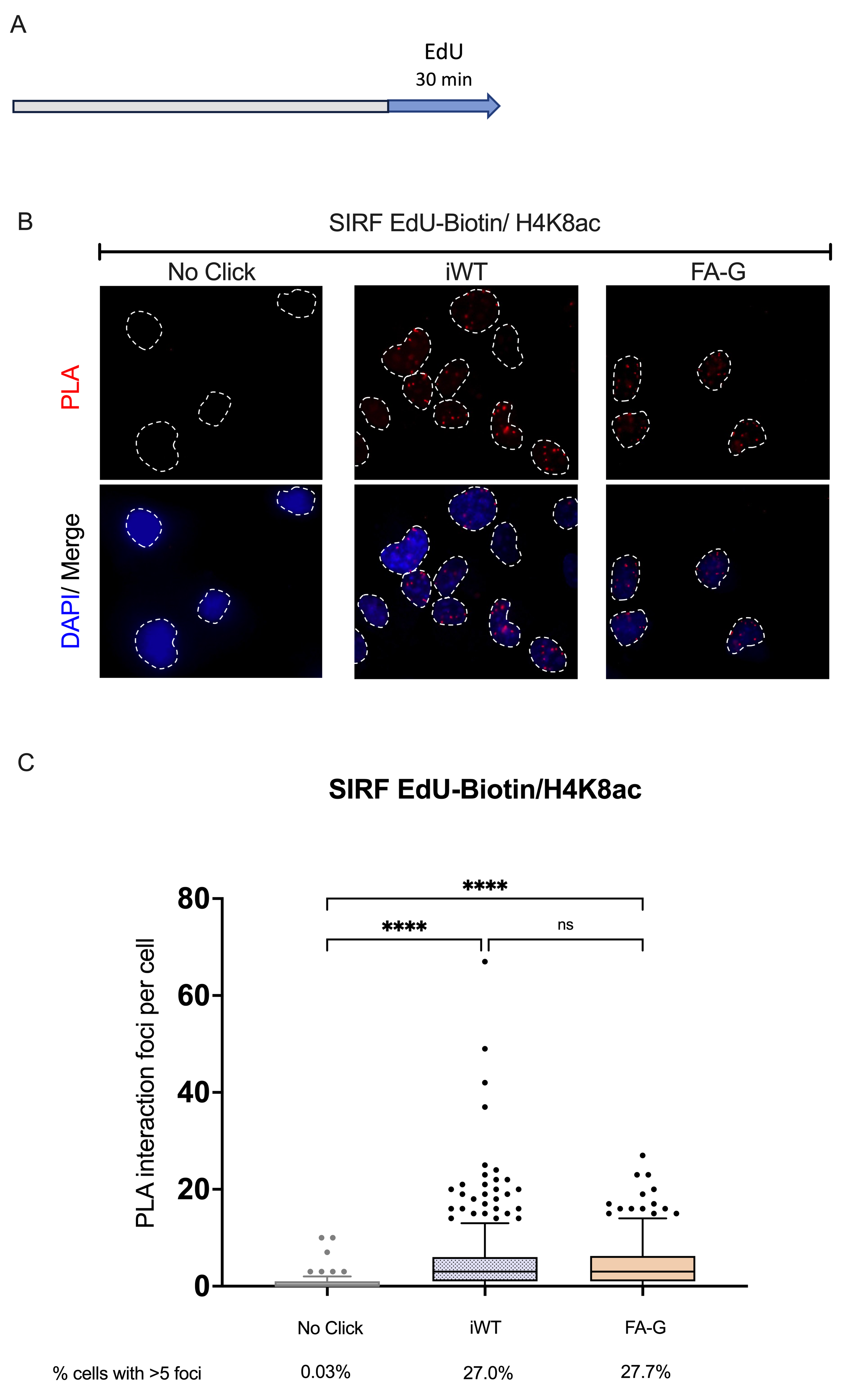

Supplement: S1 Fig — (A) FANCG proficient PD326+G (iWT) and FANCG deficient PD326+EV (FA-G) cells were seeded in coverslips and labeled the next day (grey bar) with EdU [10 μM] for 30 min and fixed (blue arrow), EdU was then clicked to biotin azide before performing a PLA. (B) Representative images of a SIRF assay showing PLA foci of the interaction between EdU-Biotin and H4K8ac, dotted lines mark the nuclei. (C) Quantification of the PLA interaction foci per cell from one independent replicate, a no click sample was included to ascertain PLA background signal. Cells were treated as in S1A Fig. At least 200 cells per condition were analyzed. Differences were probed using the Kruskal Wallis test with Dunn’s post-test for multiple comparisons. ****p<0.0001; ns 0.1234. Data are represented as mean ± SEM. (TIF) [file pone.0298032.s001.tif]

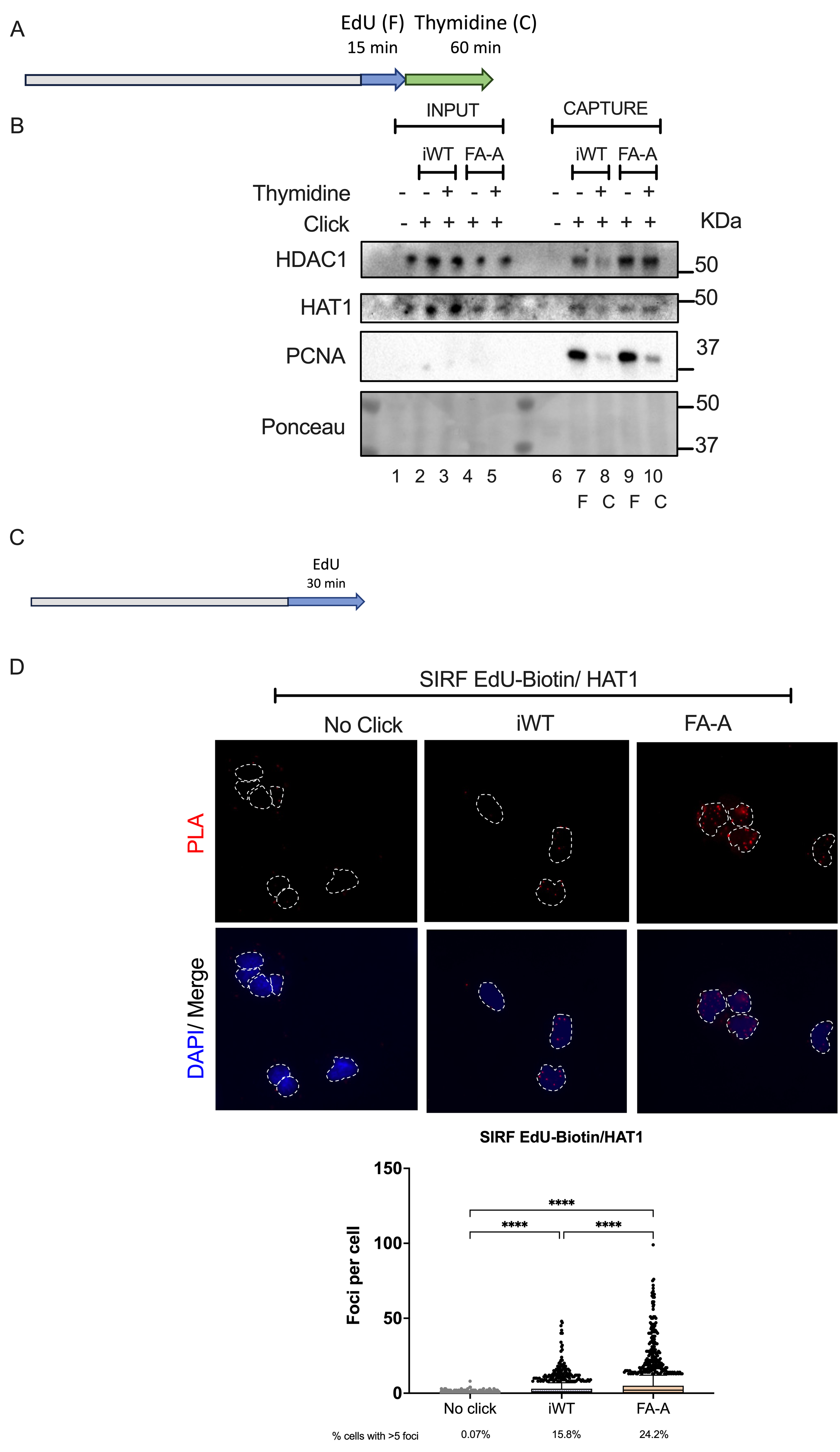

Supplement: S2 Fig — (A) For aniPOND assays, cells were seeded and labeled the next day (grey bar) with an EdU [10 μM] pulse the last 15 min of culture (blue arrow), harvested immediately or chased in thymidine for 1 hour before harvest (green arrow). The cells were then clicked to biotin-azide before lysis and incubated overnight with streptavidin beads to precipitate EdU-associated proteins. (B) Representative immunoblots after capture of the replication fork in iWT and FA-A cells. Lanes 1 to 5 total protein of INPUT samples: lane 1 no click control, iWT cells in lanes 2 (pulse) and 3 (pulse and chase), FA-A cells in lanes 4 (pulse) and 5 (pulse and chase). Lanes 6 to 10 CAPTURE samples: lane 6 no click control, iWT cells in lanes 7 (pulse) and 8 (pulse and chase), FA-A cells in lanes 9 (pulse) and 10 (pulse and chase). All blots were performed in the same membrane for which a section of the Ponceau dye is shown. Blots show the presence of histone deacetylase 1 (HDAC1) and histone acetyl transferase 1 (HAT1). Cells were treated as in S2A Fig. F: capture of replication fork, C: capture of mature chromatin. (C) For SIRF assay, cells were seeded in coverslips and labeled the next day (grey bar) with EdU [10 μM] for 30 min and fixed (blue arrow), EdU was then clicked to biotin azide before performing a PLA. (D) Representative images of a SIRF assay showing PLA foci of the interaction between EdU-biotin and HAT1 (top), dotted lines mark the nuclei. Quantification of PLA interaction foci per cell from three independent replicates (bottom), a no click sample was included to ascertain PLA background signal. Cells were treated as in S2C Fig. At least 500 cells per condition per experiment were analyzed. Differences were probed using the Kruskal Wallis test with Dunn’s post-test for multiple comparisons. ****p<0.0001. Data are represented as mean ± SEM. (TIF) [file pone.0298032.s002.tif]

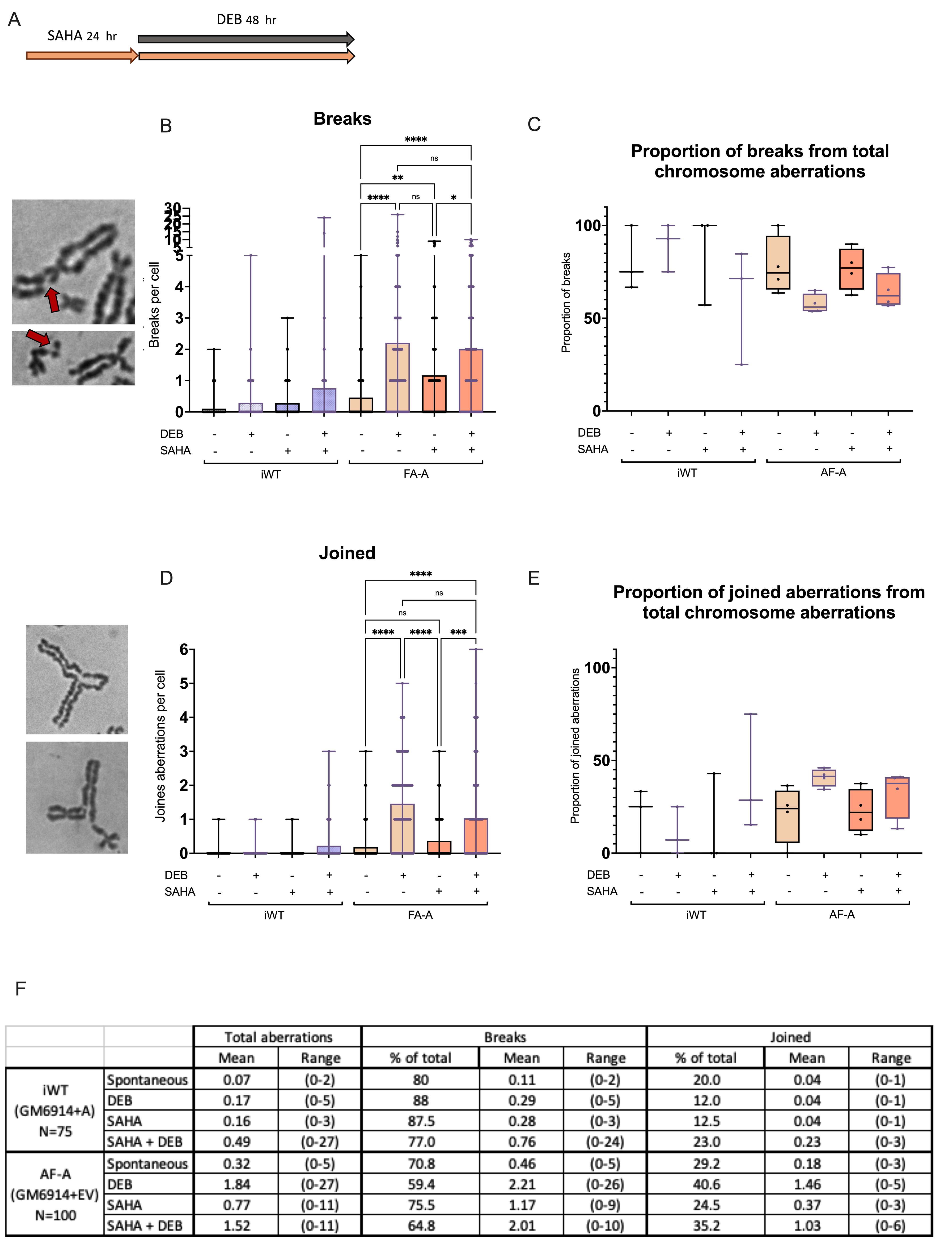

Supplement: S3 Fig — (A) For chromosome breakage analysis cells were pre-treated with SAHA [1 μM] for 24h (orange arrow) before inducing chromosomal aberrations with DEB [5 ng/mL] and the culture was continued for additional 48h (dark gray arrow). In cultures treated with SAHA the total time of exposure before harvest was 72 hours. (B) Metaphase spread with representative breaks, DNA damage that affects the continuity of the metaphasic chromosome affecting either single or both chromatids (Left panel). Quantification of chromosomal breaks per cell shows a significant increase of breaks in FA-A cells when treated with DEB, SAHA or a combination of both agents (Right panel). Cells were treated as in S3A Fig. Three (iWT) or four (FA-A) independent experiments were performed. Mean and range are depicted. Differences between groups were probed using the Kruskal Wallis test. ****p<0.0001; **0.0021; *0.0332; ns 0.1234. (C) Proportion of chromosomal breaks from total chromosomal aberrations. Mean and range of three (iWT) or four (FA-A) independent experiments are graphed. SAHA mainly induces breaks in FA-A cells. (D) Metaphase spread with representative joined aberrations, resulting from breaks that attach to an incorrect partner, particularly radial figures, the classic aberration in FA/BRCA deficient cells (Left panel). Quantification of joined chromosomal aberrations per cell shows an increase in joined aberrations upon exposure to DEB in FA cells (Right panel). Cells were treated as in S3A Fig. Three (iWT) or four (FA-A) independent experiments were performed. Mean and range are depicted. Differences between groups were probed using the Kruskal Wallis test. ****p<0.0001; ***p<0.0002; ns 0.1234. (E) Proportion of joined chromosomal aberrations from total chromosomal aberrations. Mean and range of three (iWT) or four (FA-A) independent experiments are graphed. SAHA pre-treatment appears to reduce the proportion of joined aberrations induced by DEB. (F) Table showing median and range of chromo [file pone.0298032.s003.tif]
