# Supplementary material for: Reversion from basal histone H4 hypoacetylation at the replication fork increases DNA damage in FANCA deficient cells
Source: PLoS One. 2024 May 31;19(5):e0298032. doi: 10.1371/journal.pone.0298032 (PMC11142588; doi:10.1371/journal.pone.0298032)
Supplement: Attachment — Submitted filename: Response to reviewers Garcia de Teresa .docx [file pone.0298032.s005.docx]

December 28^th^, 2023

Garcia de Teresa PLOS ONE manuscript “Reversion from basal histone H4 hypoacetylation at the replication fork increases DNA damage in FANCA deficient cells”

Rebuttal letter Point-by-point response to reviewers from PLOS GENETICS

**Editor,**

PLOS ONE

To the Editor,

Thank you for considering the transfer to PLOS ONE and the valuable reviews performed while under consideration at PLOS Genetics of our manuscript entitled “Reversion from basal histone H4 hypoacetylation at the replication fork increases DNA damage in FANCA deficient cells”.

Overall, we are pleased with the positive comments of the reviewers, but we agree that the manuscript could be improved. We have used the reviewers’ well thought comments to address their raised concerns and re-write some sections of the manuscript.

We have also revised all Figures and added two new supplemental figures to include some of the additional experiments suggested by reviewer 1.

**1- Revised** **Figure 1.**

FANCA WB blots from **revised Figures 1B and 1C** (Reviewer # 2 and # 3), were taken away from this figure and blots for H4 were added as loading controls (Reviewer # 2).

Following Reviewer # 1’s comment, we changed the graphs of **Revised figures 1E and 1F**, to depict a representative assay including no click condition which amounts to a single antibody control to ascertain background signal (Reviewer # 1).

2-**New** **Supplementary Figure** **1**

In this new figure, we show that in *FANCG* deficient fibroblasts and its isogenic counterpart, there is no difference in the H4K8 acetylation status, as ascertained by PLA (Reviewer # 1).

3- **Revised Figure 2**

In **revised Figure 2B**, the FANCA WB blot (Reviewer # 2 and # 3), was taken away from this figure and blot for H4 was added as loading control (Reviewer # 2).

Also, **Revised figure 2D** was modified to show a representative assay including the no click condition control to ascertain background signal data (Reviewer # 1).

4- **New Supplementary Figure 2**

In this new figure we provide additional data to support HDAC1 enrichment at the FA-A fork (Reviewers # 1, # 2 and # 3), as well as data on HAT1 and its association to the replication fork (Reviewer # 2).

4- **Revised Figure 3**

In **Revised Figure 3B**, the H4 blot was added as a loading control (Reviewer # 2), and the graph illustrating 3E was changed (Reviewer # 2).

5- **New revised supplemental Figure 3**

This figure corresponds to what was presented as supplemental Figure 1 in the earlier version of this manuscript.

4- **Revised Figure 4**

In **Revised Figure 4D**, the H4 blot was added as a loading control (Reviewer # 2).

We believe that our findings are sound and describe an interesting phenotype in Fanconi anemia cells.

We have attached a **Point-by-Point** response to the reviews below.

With these changes, we believe that our manuscript has significantly improved. Thank you again for considering our paper for publication in *PLOS ONE*.

Sincerely yours,

Sara Frías and Benilde García de Teresa

**Point-by-Point Response to the Reviewers’ comments**: (Our Responses are in *italics. We have also inserted the relevant new text and the new references into the manuscript.*)

**Reviewer #1**: The manuscript by Garcia de Teresa et al. evaluates the histones acetylation status of replication forks from FANCA deficient cells using aniPOND and SIRF assays. They found hypoacetylated histones H3 and H4 in FANCA deficient replication forks (RFs) concomitant to increased association of HDAC1. Chemical inhibition of HDACs in FANCA cells restores H3/H4 acetylation status to levels similar to those observed in FANCA proficient cells, but induced deleterious effects measured as DNA damage, G2 cell cycle arrest, reduced RAD51 foci and increased RPA-S33 signal.

The overall findings described in the MS are novel and original to the Fanconi Anemia and replication fork biology fields, and the description of hypoacetylation of histones in FANCA cells could be of interest for a general audience. The research is scientifically sound, however, some data presented in the manuscript lack some control conditions, some complementary experimental approaches are needed to support author´s statements, and text reformatting is requested before publication in PLoS Genetics Journal.

*R= We are pleased by the favorable comments from this reviewer and appreciate their constructive recommendations.*

**Major points**:

-The entire set of data is obtained using a GM6914+EV (FANCA deficient) vs GM6914+FA-A complemented cell lines, as the authors pointed out at the limitations of the study. As it stands, it is difficult to ascertain if hypoacetylated H3/H4 and associated phenotypes observed in FANCA deficient cells are FANCA-, FA-pathway or any replication fork stability gene´s specific. Knockdown or knockout assays of other FA, BRCA and/or other unrelated gene functioning at replication fork should be tested to reinforce their conclusions and convey their message to a broader audience. Authors could employ H4K8ac SIRF as a redout, or any other means to address this reviewer´s concern.

*R= Following this reviewer’s suggestion to use SIRF as a readout, we analyzed H4K8ac status at the replication fork of FANCG deficient PD326+EV cells and its isogenic counterpart PD326+G, these data are presented in the* ***new supplemental Figure 1****.*

-The SIRF assay is a very sensitive technique to detect interaction between molecules in fixed cells. Due to the extreme sensitivity of the amplification reaction it might sometimes leads to wrong conclusions when appropriate controls are absent. For this reason, I would suggest the authors to include the single antibody controls (EdU-Biotin, HDAC1 and H4K8ac single controls) to ascertain the background signal.

*R= We thank the reviewer for this thoughtful comment, they are absolutely right to point out that the data from this kind of controls were missing from the original submission. We have modified* ***Figures 1E, 1F and 2D*** *to include the data from the “no click” condition performed contemporaneously. We considered this a good control since it not only ascertains that the observed interaction is specific to the molecules being probed, but in this context, it also ascertains that the interactions detected correspond to those occurring at replication forks.*

Moreover, there must have been a mistake in the table describing antibodies. Anti-HDAC1 (ab7028), anti-H4K8ac (GTX60906) and anti-Biotin (cat D5A7) were all raised in rabbits. This concern must be addressed.

*R= We apologize for this mistake. We have now made the appropriate correction in the table describing the antibodies.*

-There is some inconsistencies in the aniPOND data related to HDAC1. Figure 2 shows that FANCA replication forks accumulated HDAC1, suggesting that hypoacetylated H3/H4 observed in FANCA cells could be due to this fact, and this was confirmed by SIRF assay. However, figure 3 shows equal HDAC1 amounts in wildtype and FANCA cells.  Because western blot is a semi-quantitative technique, I suggest the authors confirm the measurements obtained by SIRF by other technique.

*R= We thank the reviewer for this comment. To strengthen our claim, we have added supplementary figure 2 where we show aniPOND data from an experiment exploring the fork and mature chromatin, where enrichment of HDAC1 in FANCA deficient samples is shown.*

-Several reports in the literature suggest that HDAC3 play a role in replication fork dynamics, in addition to HDAC1 (Kehrli et al. 2016; Agudelo-Garcia et al 2020;Conti et al 2010). What was the reasoning behind blotting for HDAC1 instead of HDAC3 upon aniPOND? Does HDAC3 protein follow the same trend as HDAC1? Could HDAC3 be responsible for the phenotypes described here, or at least contribute somehow? This should be described in the MS. Due to SAHA being a HDACs paninhibitor, it would be interesting to address the contribution of HDAC1 or HDAC3 separately to the observed phenotypes. HDAC1 association to replication forks might not be responsible for the phenotypes observed by SAHA. For this reason, the authors should use a HDAC1 specific inhibitor (e.g. CBUD-1001) to ascertain the direct contribution of HDAC1. Alternatively, SIRF assays using siHDAC1 or siHDAC3 could be very informative.

*R= We thank the reviewer for this valuable comment. The reasoning behind evaluating HDAC1 was indeed the fact that this deacetylase has consistently been shown to be present at the replication fork, a feature that has not been shown for HDAC3. As the reviewer points out, other deacetylases, including HDAC3, may be contributing to the phenotypes that we observed. We have added this particular information to the main text in the “limitations section”. Unfortunately, neither specific HDAC inhibitors nor siRNAs were available to us.*

-The authors should explain the use of different statistical tests for similar experimental approaches (Mann Whitney, Kruskal Wallis, t student, Dunn test…). Are the authors comparing the mean or the median of two conditions, or several conditions at the same time? This should be clarified.

*R= The reviewer is kind to note this is not clear. For all comparisons made, we verified the data distribution. If the distribution was normal, the means were compared using One way ANOVA with Tukey’s post-test for multiple comparisons. When only two groups were compared, we used a student’s t test. And when the distribution was not normal, we used the Kruskal Wallis non parametric test with Dunn’s post-test for multiple comparisons. This was clarified in the methods section and figure captures.*

**Minor points**:

-The discussion should include a paragraph about the biological relevance of replication fork associated H3/H4 hypoacetylation in the absence of FANCA. Is this a reflect of impaired histone modification machinery in FANCA cells? Is this fact a compensatory mechanism to decreased active transcription thus avoiding Transcription-replication conflicts? Is it due to compensatory mechanisms only to recruit specific nucleases or the specific nucleases recruitment is just a consequence of aberrant fork structure?

*R= We thank the reviewer for this thoughtful and generous comment. As suggested, we have added a paragraph in the discussion section touching on these subjects (page # 19).*

- References are formatted in Spanish language. Please reformat references accordingly.

*R= We thank the reviewer for spotting this and apologize for the inattention. It has now been formatted in English.*

- Pg 32 Ln 553. Erase sentence.

*R= We again regret the inattention and have now erased this sentence*

**Reviewer #2**: In this manuscript, Teresa et al. examined histone H4 acetylation status at replication fork in FANCA deficient cells. The authors used two methods, accelerated native immunoprecipitation of nascent DNA (aniPOND) and in situ protein interactions in the replication fork (SIRF), and found decreased H4 acetylation at elongating replication fork in FANCA deficient cells. They next used an HDAC inhibitor, SAHA, to treat the cells and showed that SAHA increased H4 acetylation, but at the cost of DNA damage and replication stress. 

Although a wide range of data are presented, the manuscript does not have a clear scientific question for the authors to address. The lack of a key question or hypothesis makes the manuscript boring and hard to understand. The data do not offer sufficient mechanistic insights to address functions of the FA pathway in DNA replication. Why is it important to evaluate H4 acetylation status in FANCA deficient cells? How could the FANCA deficiency impact H4 acetylation at the replication fork? There is little description of the rationale throughout the manuscript.

*R= We thank the reviewer for these comments. Considering their observations, we have modified the “introduction section” (page # 5) to explicit the question and added a paragraph in the “discussion section” to highlight the relevance of evaluating histone acetylation status in FA-A cells (page # 19).*

Another major concern is no valid loading control is used for the aniPOND experiments. The Ponceau staining cannot serve as a good control. Western blots with histone antibodies such as H3 or H4 can provide better loading information.

*R= We appreciate this reviewer’s comment, although the protein community has recently been discussing the parity or even superiority of total protein as a loading control when compared to houskeeping proteins (1,2). In recognition of the reviewer’s concern, we have added the H4 blots for all presented aniPOND experiments.*

1.	Moritz CP. Tubulin or Not Tubulin: Heading Toward Total Protein Staining as Loading Control in Western Blots. PROTEOMICS. 2017;17(20):1600189.

2.	Sander H, Wallace S, Plouse R, Tiwari S, Gomes AV. Ponceau S waste: Ponceau S staining for total protein normalization. Anal Biochem. 2019 Jun 15;575:44–53.

**Some specific comments**:

1) The FANCA Western blot data in Figure 1 and 2 is not convincing. It is hard to tell if that’s a specific band.

*R= The reviewer is kind to note this. The depicted blots are the clearer images we were able to obtain. Given that the blot data is not convincing and that FANCA deficiency of GM6914 cells is well established (3) we have eliminated the blots from the figures.*

3.	Näf D, Kupfer GM, Suliman A, Lambert K, D’Andrea AD. Functional Activity of the Fanconi Anemia Protein FAA Requires FAC Binding and Nuclear Localization. Mol Cell Biol. 1998 Oct 1;18(10):5952–60.

2) The HDAC1 aniPOND presented in Fig. 2B is interesting, because it can potentially explain why H4 acetylation is depleted in FANCA replication forks. But the HDAC1 Western blot data is not convincing. It is also possible the depletion of H4 acetylation is due to lack of HATs associated with replication fork. Why only chose HDAC1?

*R= We agree with the reviewer that the HDAC1 data at the FANCA deficient replication fork is interesting. To strengthen our claim, we have added supplementary figure 2 where we show aniPOND data from an experiment exploring the fork and mature chromatin, where enrichment of HDAC1 in FANCA deficient samples is shown. In this experiment we also blotted HAT1 but did not find evidence of its reduction in FA-A cells. On the contrary, SIRF data, also presented in Sup Fig 2, evidences enrichment of HAT1 at the FA-A replication fork. Levels of PCAF/KAT2B can also be seen in Figure 4D, the FA-A fork is enriched in this protein when compared to iWT cells.*

3) Why H2AX phosphorylation is not induced by SAHA in WT cells (Figure 3E)?

*R= We thank the reviewer for his careful revision of our manuscript. We performed two additional replicates of this assay that recapitulate the increase in H2AX phosphorylation upon inhibition of HDAC in both FA-A and iWT cells, and have changed the panel of figure 3E.*

4) The statement on page 10, line 202, “MRE11, a nuclease responsible for fork degradation (24,25) is more abundant in FA-A deficient replication forks.” Where is the data?

*R= The reviewer is right to note that there is no data supporting this statement which is the result of an error in the manuscript. We thank the reviewer for spotting this, it has now been corrected.*

**Reviewer #3**: This m/s describes analysis of histone acetylation marks at replication forks in FANCA deficient cells. The data suggests that chromatin is hypoacetylated in FANCA mutants in chromatin, including at chromatin captured by iPOND (Fig 1B) or visualized by proximity ligation assay (PLA, Fig 1F). It is difficult to conclude that the observed hypoacetylation is restricted to ongoing replication forks, since the input samples shown for the iPOND experiments, as well as the fork-associated samples, reveal reductions in histone acetylation in FANCA mutants. This observation is compatible with previous findings (e.g., PMID 26446986, not referenced in the current m/s).

*R= We thank the reviewer for their comments. As noted by them, the observation of reduced acetylation in FANCA deficient cells in the input samples is indeed compatible with data presented by Renaud et al showing basal hypoacetylation of siRNA mediated FA pathway deficient cells. We have noted this in the manuscript and added the appropriate reference (page # 19). We have also changed the manuscript to point that this acetylation impairment is notably important at replication forks, a structure that is expected to be richly acetylated.*

As a corollary, the authors report increased HDAC signals in FANCA mutant cells, using iPOND and PLA. Some of the western blot data is unconvincing (e.g., FANCA iPOND data, HDAC iPOND data) and not all of the data is supported by statistical analysis (e.g., Fig 2D).

*R= The reviewer is kind to note this. Given that FANCA deficiency of GM6914 cells is well known (3) we have eliminated the FANCA blots from the figures. With respect to HDAC1 data, we have performed an additional assay and present the data supported by statistical analysis for Fig 2D, and added supplementary Figure 2 where we present more aniPOND data revealing HDAC1 enrichment.*

3.	Näf D, Kupfer GM, Suliman A, Lambert K, D’Andrea AD. Functional Activity of the Fanconi Anemia Protein FAA Requires FAC Binding and Nuclear Localization. Mol Cell Biol. 1998 Oct 1;18(10):5952–60.

The authors find that treatment of either WT or FANCA mutant cells with the HDAC inhibitor SAHA destabilizes the genome, suggested by the accumulation of cells in G2 and the appearance of a subdiploid population of cells by flow cytometry (Fig 3D). This effect is seen in both WT and FANCA mutant cells, but is more pronounced in FANCA mutants. Inhibition of HDAC is a very blunt tool for this investigation. One reading of the data is that HDAC has similar effects in both cell types, but the defect in the FA pathway in FANCA mutants exaggerates the impact on cell viability.

*R= We thank the reviewer for their comment. We agree that HDAC inhibition is a blunt tool, but it was the one available to us. We also concur with the reviewer regarding the fact that HDAC inhibition has similar effects in both cell lines being more severe in FA-A cells. We have modified the Figure 3 caption to convey this.*

Overall, the findings reported here appear limited in novelty and may lack the impact needed for PLoS Genetics.

*R= The reviewer judges the findings presented here as limited in novelty, and refers to the paper of Renaud et al, where although they discuss the effect of hypoacetylation of H4K16 in response to MMC damage, they fail to discuss basal hypoacetylation produced by deficiency of FA pathway genes. Although histone acetylation impairment of H4K16 has been previously reported in FA pathway deficient cells (4), the observations made here show that this impairment touches other residues that have not been explored before, and support the notion that the FA pathway is important for chromatin conformation and genome stability. Moreover, we show that altering the new found balance of histone acetylation at the FA-A replication fork results in DNA damage.*

4.	Renaud E, Barascu A, Rosselli F. Impaired TIP60-mediated H4K16 acetylation accounts for the aberrant chromatin accumulation of 53BP1 and RAP80 in Fanconi anemia pathway-deficient cells. Nucleic Acids Res. 2016 Jan 29;44(2):648–56.
