# Supplementary material for: Reversion from basal histone H4 hypoacetylation at the replication fork increases DNA damage in FANCA deficient cells
Source: PLoS One. 2024 May 31;19(5):e0298032. doi: 10.1371/journal.pone.0298032 (PMC11142588; doi:10.1371/journal.pone.0298032)
Supplement: Peer review history

# Author comment: Author response to previous submission

**DOI:** 10.1371/journal.pone.0298032.r001

**Transfer Alert**

This paper was transferred from another journal. As a result, its full editorial history (including decision letters, peer reviews and author responses) may not be present.

4 Jan 2024

Attachment Submitted filename: Response to reviewers Garcia de Teresa .docx

# Editor report: Decision Letter 0

**DOI:** 10.1371/journal.pone.0298032.r002

**Authors:** Zhiming Li

17 Jan 2024

Reversion from basal histone H4 hypoacetylation at the replication fork increases DNA damage in FANCA deficient cells.

PONE-D-24-00154

Dear Dr. Frias,

Thank you for transferring your manuscript to PLOS ONE. Based on the comments we received from reviewers at PLOS Genetics and the revisions you've made to address them, we’re pleased to inform you that your manuscript has been judged scientifically suitable for publication and will be formally accepted for publication once it meets all outstanding technical requirements.

Within one week, you’ll receive an e-mail detailing the required amendments. When these have been addressed, you’ll receive a formal acceptance letter and your manuscript will be scheduled for publication.

An invoice for payment will follow shortly after the formal acceptance. To ensure an efficient process, please log into Editorial Manager at http://www.editorialmanager.com/pone/, click the 'Update My Information' link at the top of the page, and double check that your user information is up-to-date. If you have any billing related questions, please contact our Author Billing department directly at authorbilling@plos.org.

If your institution or institutions have a press office, please notify them about your upcoming paper to help maximize its impact. If they’ll be preparing press materials, please inform our press team as soon as possible -- no later than 48 hours after receiving the formal acceptance. Your manuscript will remain under strict press embargo until 2 pm Eastern Time on the date of publication. For more information, please contact onepress@plos.org.

Kind regards,

Zhiming Li, Ph.D.

Academic Editor

PLOS ONE

Additional Editor Comments (optional):

The authors greatly improved the manuscript by addressing most of the comments from the reviewers.

Reviewers' comments:
